# OLDER ADULTS' INDIVIDUAL TRAJECTORIES IN SOCIAL STATUS AND AGING ANXIETY

**DOI:** 10.1093/geroni/igac059.2004

**Published:** 2022-12-20

**Authors:** Tim Kuball, Georg Jahn

**Affiliations:** Chemnitz University of Technology, Chemnitz, Sachsen, Germany; Chemnitz University of Technology, Chemnitz, Sachsen, Germany

## Abstract

A high social standing in comparison to others is associated with positive psychological and health outcomes. Highest social standing is assigned to the group of middle-aged adults, hence, on average, older adults face a loss in status relative to younger age groups and relative to their former selves. Experienced and expected age-related changes in subjective social status and their association with aging anxiety have not yet received much attention in aging research. Using a new methodological approach, respondents indicated their perceived and expected social status for five points in time: 10 years ago, 5 years ago, now, in 5 years, in 10 years, which allowed for inter- and intrapersonal comparisons. They did the same for the average status of members of their age group. Early and later in old age (N = 191; range 65 – 88; MW = 73.5 years), participants expected higher losses in status than they have experienced in the past. However, low personal status in relation to others showed higher associations with aging anxiety (R2 = .16) than disadvantageous age-related intrapersonal changes (R2 = .14). Perception of a stable subjective status trajectory as well as distancing oneself from the group older adults, as in perceiving one’s personal status above the groups’ status, was related to reduced anxiety of aging. Taken together, analysis of individual status trajectories can help to gain new insights on attitudes toward aging. Implications for creating a more positive perception of aging are discussed.

